# Optimizing muscle satellite cell sources for cultured meat: anatomical origin influences cellular properties and quality attributes

**DOI:** 10.1038/s41538-026-00706-w

**Published:** 2026-01-14

**Authors:** Jeongeun Lee, Jinryong Park, Hyeonwoo La, Sanghoon Yoon, Kwonho Hong, Kwanseob Shim, Jeong Tae Do

**Affiliations:** 1 School of Advanced Biotechnology, Konkuk University, Seoul, South Korea; 2 Food Processing Research Group, Korea Food Research Institute, Wanju, South Korea; 3 Department of Animal Biotechnology, Jeonbuk National University, Jeonju, South Korea; 4 Department of Agricultural Convergence Technology, Jeonbuk National University, Jeonju, South Korea

**Keywords:** Biotechnology, Cell biology, Stem cells

## Abstract

Muscle satellite cells are integral to muscle growth and regeneration, making them critical for cultured meat production. However, the influence of anatomical origin on porcine muscle satellite cells (PMSCs) for food applications remains underexplored. Here, we compared PMSCs from neck, back, and leg muscles to identify the optimal cell source for cultured meat. Transcriptomic analysis showed region-specific gene expression, including differential *HOX* gene expression. Neck-derived PMSCs exhibited the highest proliferation, whereas back-derived PMSCs maintained the highest *PAX7* and *MYOD* expressions during long-term culture. Back-derived PMSCs also exhibited superior differentiation, forming thicker myotubes with the highest fusion index, favoring fast-twitch fibers, and showing the highest protein content. In contrast, neck-derived PMSCs favored slow-twitch fibers and displayed the lowest protein content. These findings underscore the significance of cell source selection in optimizing muscle tissue engineering for scalable cultured meat production, contributing to the advancement of sustainable and alternative food technologies.

## Introduction

Cultured meat offers an innovative solution to the environmental and animal welfare issues raised by traditional livestock farming^1–3^. Currently, the cultured meat approach relies on proliferating and differentiating satellite cells (SCs) extracted from livestock and can be further advanced through research focused on long-term in vitro culture systems and muscle tissue formation technologies. Therefore, the development of technologies to optimize proliferation, differentiation, and myotube formation is crucial for maximizing large-scale cultured meat production. One of the most fundamental ways to enhance this process is to select an optimal cell source. However, this aspect remains relatively underexplored^4^. Previous studies have suggested that SCs exhibit variations in extraction efficiency and heterogeneity, depending on the anatomical region from which they are derived^5–9^. Thus, selecting a cell source that can undergo stable and rapid expansion while maintaining its myogenic identity, and also retains significant differentiation capacity is essential for optimizing SCs proliferation and differentiation, ultimately enhancing cultured meat production efficiency.

SCs, located between the basal lamina and sarcolemma of muscle fibers, play a role in muscle growth and regeneration of damaged muscle fibers^9^. However, the function of SCs exhibits heterogeneity depending on their embryonic origin, muscle fiber type, and age^10–13^. The specific reasons for the differences in the proliferation and differentiation characteristics of SCs in various muscle regions are yet to be elucidated. Variability in SC proliferation and differentiation abilities affects not only the efficiency of cultured meat production but also the texture of the meat, as it is determined by muscle fiber composition patterns^14^. Therefore, it is essential to gain a comprehensive understanding of the characteristics of SCs derived from different muscle regions to select the most suitable cell line and optimize cultured meat production.

During embryonic development, head, neck, trunk, and limb muscles exhibit distinct differences depending on their origin^9,15,16^. The trunk and limb muscles originate from somites during vertebrate embryonic development, whereas most head muscles, including the masseter, arise from the cranial mesoderm. In addition, the origin of the neck muscles differs from that of the head muscles^17^. The neck muscles are categorized as somatic and non-somatic lineages, with somatic neck muscles being distinguished by the expression of Pax3^18^. Somatic neck muscles arise from myoblasts originating from the most anterior group of somites^19^, whereas trunk muscles arise from the epaxial portion and limb muscles from the hypaxial portion of the dermomyotome^20,21^. Moreover, the development of these muscles is regulated by distinct gene networks depending on their anatomical location^9,15,16,22^. These differences in embryonic origin give rise to distinct gene-expression programs in myogenic precursors, which not only influence SCs differentiation and regenerative potential but also contribute to the formation of functionally diverse skeletal muscle phenotypes^16,23^. Furthermore, the development of skeletal muscle is influenced by positional identity programs established during embryogenesis, mainly through region-specific *HOX* gene expression^24–27^. Among the 39 *HOX* genes, *HOXA11* and *HOXA13* primarily participate in hindlimb muscle development, and their expression has been shown to promote slow-type myogenic programs and indirectly influence adipogenic regulation^26,28^. This evidence indicates that skeletal muscle exhibits intrinsic heterogeneity shaped by its embryonic origin. Lineage-dependent positional programs, including region-specific *HOX* gene expression, contribute to distinct myogenic identities across anatomical sites. Because SCs can retain these positional signatures, their proliferative and differentiation capacities may differ by muscle of origin, potentially influencing fiber organization, metabolic characteristics, and texture relevant to cultured meat production.

Here, we extracted three sources of porcine muscle satellite cells (PMSCs) from the neck, back, and leg tissues and compared global gene expression patterns, proliferation rates, *HOX* gene expression patterns, differentiation abilities, myotube formation, and fiber type composition. We found that the anatomical origin of the PMSCs influences cell characteristics and proposed a selection strategy that can be applied during the initial stages of cultured meat production. This study provides fundamental insights into the biological and functional diversity of PMSCs and suggests a strategy for identifying the optimal cell source for sustainable meat production.

## Results

### Isolation and quantification of PMSCs by anatomical region

**Fig. 1:**
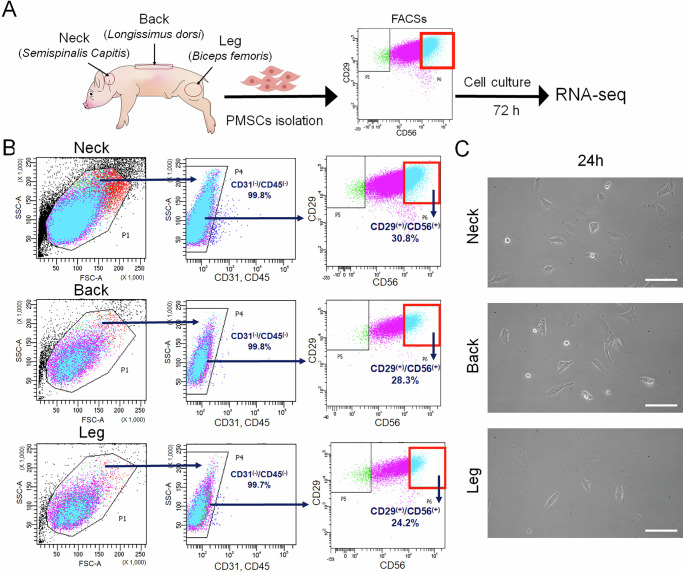
Isolation and quantification of porcine muscle satellite cells (PMSCs) from different anatomical regions. **A** Schematic representation of PMSC isolation and analysis. **B** Neck (*Semispinalis Capitis*), back (*Longissimus dorsi*), and leg (*Biceps femoris*) derived SCs were purified using FACS. The first panel shows forward scatter (FSC) and side scatter (SSC) gating for initial cell selection. The second panel depicts CD31⁻/CD45⁻ gating to exclude endothelial and hematopoietic cells. The final panel displays the percentage of CD29⁺/CD56⁺ cells, identified as PMSCs, for each region: neck (30.8%), back (28.3%), and leg (24.2%). **C** Representative phase-contrast images of PMSCs from each region after 24 h of culture. Cells from all regions exhibit an asymmetrically spindle-shaped morphology. Scale bars: 200 μm.

To characterize the anatomical differences in PMSCs, we isolated PMSCs from the neck, back, and leg tissues (Fig. 1A). These muscles were selected because they develop from different regions of the myotome and are commonly used as meat sources. Immediately following tissue dissociation, the total number of mixed cells, including all muscle-related types, was about 9.9 × 10⁵ cells/g in the neck, 1.06 × 10⁶ cells/g in the back, and 1.43 × 10⁶ cells/g in the leg (data not shown). To minimize external influences on SCs, we used FACS to purify and quantify PMSCs derived from each anatomical region. The CD31^−^/CD45^−^/CD29^+^/CD56^+^ cell population (light blue) was identified as SCs. FACS analysis revealed significant differences in the PMSC numbers, with the neck showing the highest count, followed by the back, whereas the legs exhibited the lowest number of purified cells (Fig. 1B). This result is consistent with previous reports, suggesting that the number of PMSCs varies among skeletal muscle segments within the same individual^6,7^. These results were confirmed by FACS analysis of PMSCs isolated from the neck, back, and legs of two additional LYD pigs (Fig. S1). In addition, PMSCs from each region exhibited a similar asymmetrical spindle-shaped morphology with a small and round shape (Fig. 1C).

### Transcriptomic differences among PMSCs from different anatomical regions

**Fig. 2:**
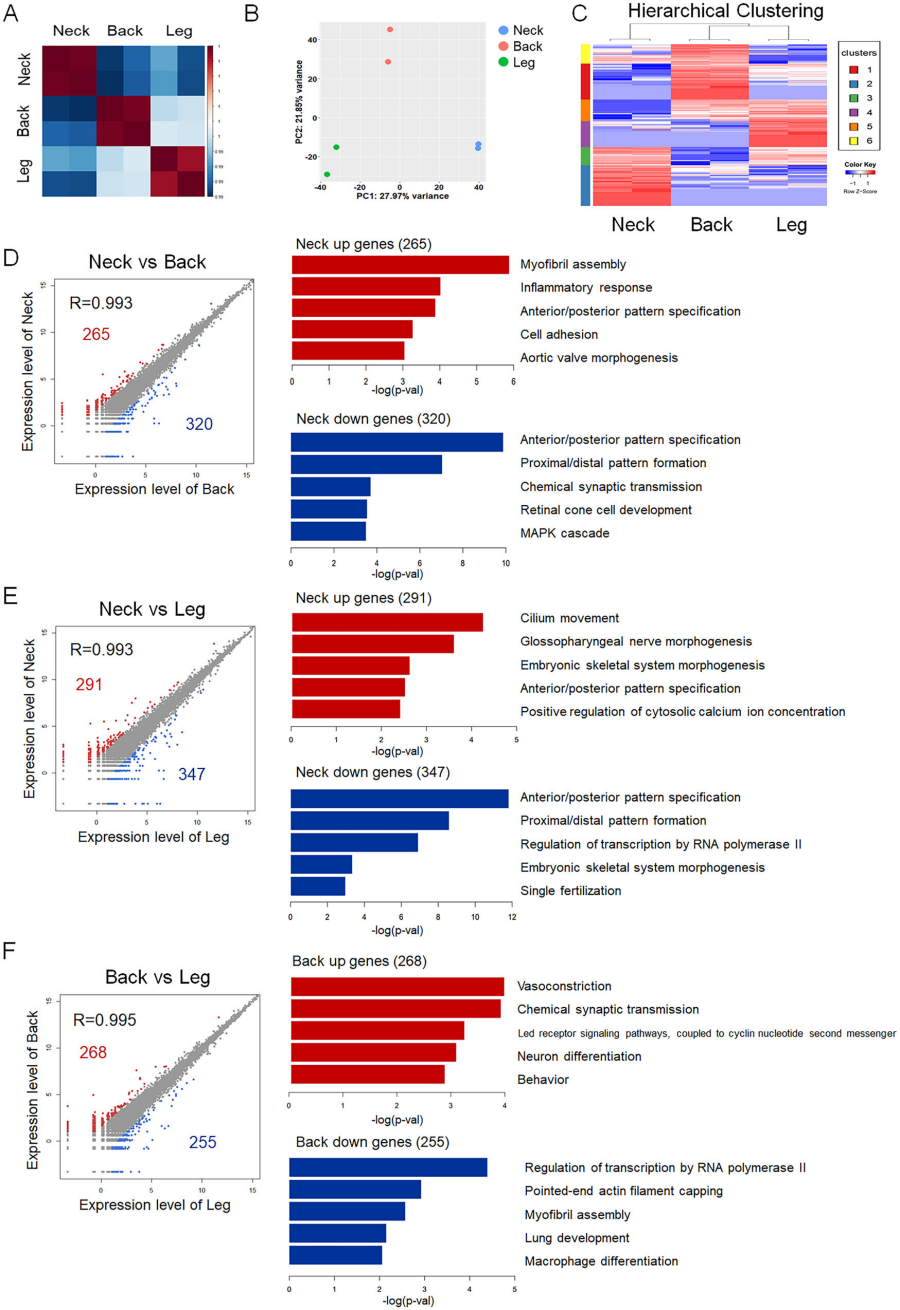
Transcriptomic analysis of PMSCs from different anatomical regions. **A** Heatmap and **B** principal component analysis (PCA) plot showing transcriptomic differences among PMSCs isolated from the neck, back, and leg muscles. The heatmap indicates hierarchical clustering of differentially expressed genes (DEGs), and the PCA plot illustrates clear separation of samples by anatomical region. PC1 and PC2 accounts for 27.97 and 21.8% of the variance, respectively (n = 2). **C** Heatmap and hierarchical clustering of DEGs across PMSCs from the neck, back, and leg regions. **D**–**F** Pairwise comparisons of gene expression patterns between anatomical regions. Scatter plots show correlation coefficients (R values) and numbers of upregulated (red) and downregulated (blue) genes in each comparison: neck vs. back (**D**), neck vs. leg (**E**), and back vs. leg (**F**). Gene ontology biological process (GOBP) analysis (bar graphs) highlights biological processes associated with upregulated (red) and downregulated (blue) genes in each comparison. n = 2; FC ≥ 3, *p*-value < 0.05, FPKM ≥ 2. FPKM; fragments per kilobase of transcript per million mapped reads.

To investigate the transcriptomic variations among PMSCs isolated from different anatomical regions, RNA sequencing analysis was conducted after 72 h of cell culture. A heatmap of the DEGs revealed distinct clustering of PMSCs derived from neck, back, and leg muscles, indicating region-specific gene expression patterns (Fig. 2A). PCA further demonstrated clear segregation among the three groups, with PC1 accounting for 27.97% of the variance and PC2 accounting for 21.8%, suggesting significant transcriptomic differences based on anatomical origin (Fig. 2B). Hierarchical clustering analysis confirmed that PMSCs from each region exhibited unique transcriptomic signatures with genes clustered into distinct groups corresponding to their anatomical origins (Fig. 2C). In total, 644 DEGs were categorized into six distinct clusters: Cluster 1 (144 genes), Cluster 2 (163 genes), Cluster 3 (74 genes), Cluster 4 (104 genes), Cluster 5 (87 genes), and Cluster 6 (77 genes). To identify the biological processes (BP) represented in each cluster, GO term enrichment analysis was conducted using DAVID with *p*-value ≤ 0.05 (Fig. S2). To further ensure the reliability of the categories, we presented terms with a false discovery rate (FDR) ≤ 0.05 (Table 1). No terms in clusters 3, 4, or 6 met the FDR threshold. However, the most abundant terms in clusters 1, 2, and 5 were associated with embryonic development, including anterior/posterior pattern specifications, proximal/distal pattern formation, and embryonic skeletal muscle system morphogenesis.

**Table 1:** Gene ontology (GO) analysis of biological process (BP) terms was identified for each cluster using DAVID with *p*-value < 0.05 and FDR < 0.05

Cluster	GO ID	Term	Count	%	*p*-value	FDR
1	GO:0009952	Anterior/posterior pattern specification	6	4.28571	0.00002	0.00846
	GO:0009954	Proximal/distal pattern formation	4	2.85714	0.00012	0.02284
	GO:0006357	Regulation of transcription from RNA polymerase II promoter	15	10.71429	0.00038	0.04695
2	GO:0048704	Embryonic skeletal system morphogenesis	5	2.92398	0.00003	0.01340
	GO:0009952	Anterior/posterior pattern specification	6	3.50877	0.00005	0.01340
	GO:0021615	Glossopharyngeal nerve morphogenesis	3	1.75439	0.00012	0.02210
	GO:0030878	Thyroid gland development	4	2.33918	0.00023	0.03009
5	GO:0009952	Anterior/posterior pattern specification	7	7.29167	0.00000	0.00004

*FDR* false discovery rate.

To further assess transcriptional differences, pairwise comparisons of gene expression levels were performed between the anatomical regions (Fig. 2D–F). A total of 585 DEGs were identified between the neck and back, with 265 and 320 genes upregulated in the neck and back, respectively. GOBP term analysis revealed that the genes upregulated in the neck were primarily associated with myofibril assembly, inflammatory responses, and cell adhesion, whereas those upregulated in the back were enriched in anterior/posterior pattern specification, MAPK cascade signaling, and chemical synaptic transmission (Fig. 2D). A total of 638 DEGs were identified between the neck and leg, with 291 and 347 genes upregulated in the neck and leg, respectively. Genes upregulated in the neck were associated with cilium movement, glossopharyngeal nerve morphogenesis, and embryonic skeletal system morphogenesis, whereas those upregulated in the legs were associated with anterior/posterior pattern specification, transcriptional regulation, and single fertilization processes (Fig. 2E). A total of 523 DEGs were identified, with 268 upregulated genes in the back and 255 upregulated genes in the leg. Genes upregulated in the back were involved in vasoconstriction, chemical synaptic transmission, and neuronal differentiation, whereas those upregulated in the legs were enriched in actin filament capping, lung development, and macrophage differentiation (Fig. 2F).

The observed transcriptomic differences suggested that PMSCs from different anatomical regions exhibited distinct gene expression patterns associated with muscle function and development. PMSCs from the neck were enriched in genes related to myogenic differentiation and structural organization, whereas back-derived PMSCs exhibited gene signatures associated with neural and synaptic activity. Leg-derived PMSCs exhibit expression patterns associated with developmental processes and immune responses. These findings highlight the anatomical specificity of PMSCs and suggest that intrinsic molecular differences may contribute to functional variations in muscle development and regeneration in different muscle regions.

### *HOX* gene expression profiles in PMSCs from different anatomical regions

**Fig. 3:**
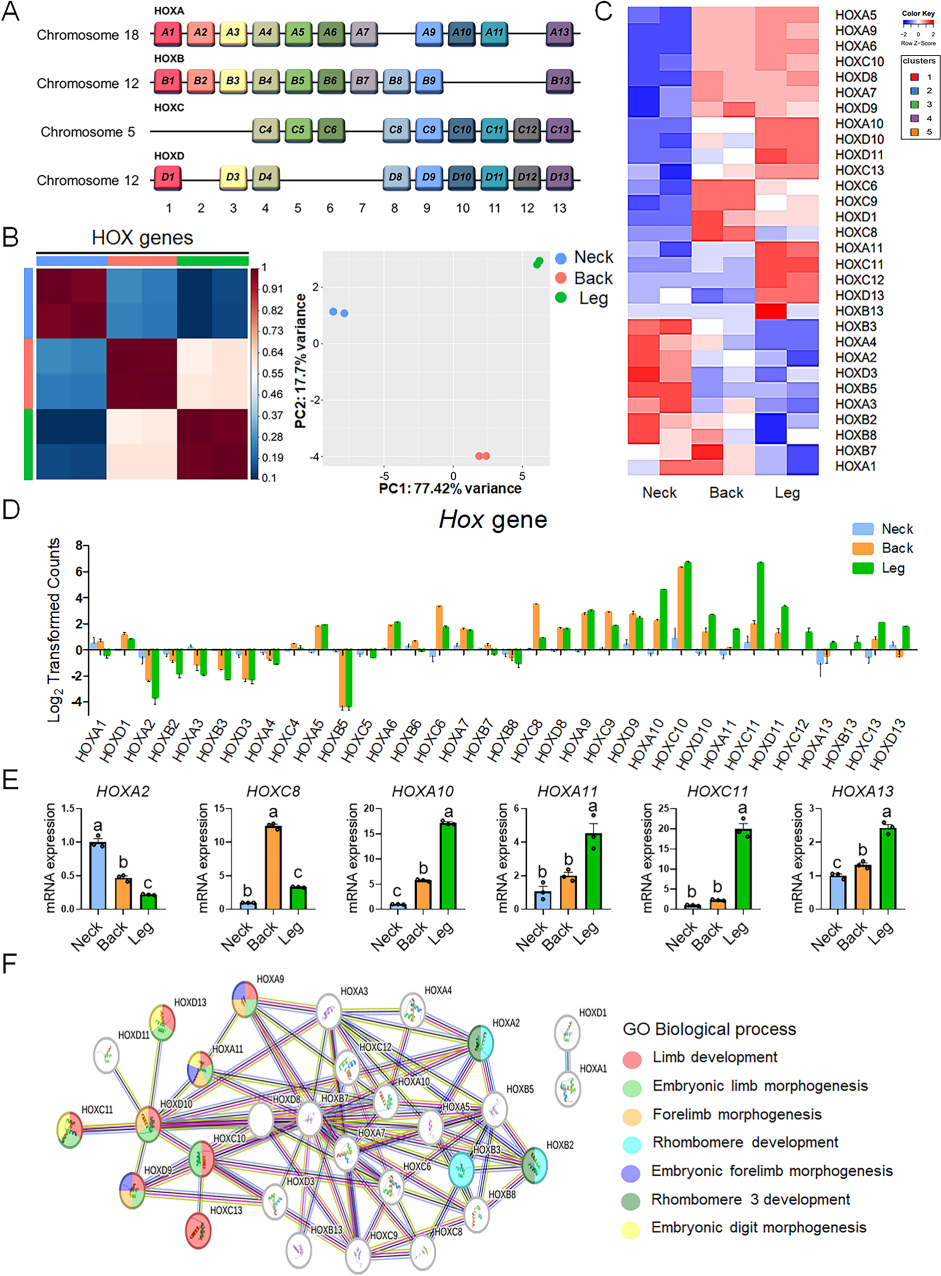
Transcriptional patterns of *HOX* genes among the neck, back, and leg-derived PMSCs. **A** Schematic representation of the *Hox* gene clusters organized by chromosomal location and paralogous groups. *Hox* genes with the same number across different clusters are color-coded to indicate their paralogous groups. Information on the *HOX* gene cluster and 13 paralogous groups in pigs was compiled based on the National Center for Biotechnology Information (NCBI) and UniProt database. **B** Heatmap (left) and PCA plot (right) showing the expression patterns of *HOX* genes across PMSCs isolated from the neck, back, and leg regions. **C** Heatmap of differentially expressed *HOX* genes in PMSCs derived from the neck, back, and leg regions. **D** Region-specific expression of *HOX* genes in different regions of PMSCs. Neck-derived PMSCs (blue), back-derived PMSCs (orange), and leg-derived PMSCs (green) exhibit region-specific *HOX* gene expression patterns. **E** PCR validation of representative region-specific *HOX* genes identified by RNA-seq across the three anatomical groups. n = 3. Statistically significant differences among the groups were indicated by different letters in Duncan’s multiple range test: lowercase letters (^a–c^) for *p* < 0.01. **F** Gene interaction network of *HOX* genes, illustrating their functional relationships. The network nodes serve as representations of genes. The edges represent associations between proteins. The network is color-coded based on GOBP terms. The network visualization was generated using the STRING database. All data are represented as mean ± SE. n = 2; *p* < 0.05, FC ≥ 3.

To examine the role of *HOX* genes, which are essential for embryonic and organ development, in PMSCs isolated from different anatomical regions, we analyzed the expression patterns of *HOX* gene clusters (*HOXA*, *HOXB*, *HOXC*, and *HOXD*) and their potential functional implications (Fig. 3). We observed notable distinctions in the *HOX* gene expression profiles of PMSCs from the neck, back, and leg regions, particularly in relation to axis-specification-related processes that are critical for embryonic development. The *HOX* genes comprise a total of 39 genes, categorized into 13 paralogous groups and organized into four clusters: Cluster A, B, C, and D (Fig. 3A)^24–27^. A higher number of paralogous groups indicate the development of more posterior regions of the body axis^26^. Therefore, we closely examined the *HOX* gene expression profiles in PMSCs from different anatomical origins to determine whether these distinct expression patterns were maintained across regions under the same culture conditions. Correlation matrix and PCA analysis indicated that *HOX* gene expression contributed significantly to the regional identity of PMSCs (Fig. 3B). In addition, a heatmap analysis showed that the expression levels of specific *HOX* genes varied across different anatomical regions (Fig. 3C). Moreover, *HOX* gene expression showed distinct patterns among PMSCs derived from the neck, back, and leg regions (Fig. 3D). Neck-derived PMSCs exhibit higher expression levels of *HOXA2*, *HOXB2*, *HOXA3*, *HOXB3*, *HOXD3*, and *HOXA4*, which are involved in cranial and cervical development. By contrast, back-derived PMSCs displayed prominent expression of *HOXA5*, *HOXA6*, *HOXC6*, *HOXA7*, *HOXC8*, *HOXD8*, *HOXA9*, and *HOXC10*, whereas leg-derived PMSCs exhibited high expression of a broader set of *HOX* genes, including *HOXA5*, *HOXA6*, *HOXA7*, *HOXD8*, *HOXA9*, *HOXD9*, *HOXA10*, *HOXC10*, *HOXD10*, *HOXA11*, *HOXC11*, *HOXD11*, *HOXC12*, *HOXB13*, *HOXC13*, and *HOXD13*, which are primarily associated with trunk and limb development. To verify the region-specific *HOX* gene signatures identified by RNA seq, we conducted RT qPCR, which consistently confirmed differential expression patterns among the three anatomical groups (Fig. 3E).

To investigate the biological functions of the differentially expressed *HOX* genes, we further analyzed the *HOX* genes using the STRING network (Fig. 3F). The STRING analysis revealed that all differentially expressed *HOX* genes, except *HOXA1* and *HOXD1*, were biologically interconnected. Genes that were highly expressed in the leg group, *HOXA9*, *HOXD9*, *HOXC10*, *HOXD10*, *HOXA11*, *HOXC11*, *HOXC13*, and *HOXD13* were associated with GO terms related to limb development, embryonic limb morphogenesis, embryonic forelimb morphogenesis, and embryonic digit morphogenesis. These results demonstrate that *HOX* genes exhibit distinct expression patterns in PMSCs from different anatomical regions, likely reflecting their roles in regional muscle development and specialization. *HOX* genes play a critical role in defining the anatomical identity of PMSCs and regulating region-specific muscle development.

### Regional differences in SC characteristics and myogenic potential during culture

**Fig. 4:**
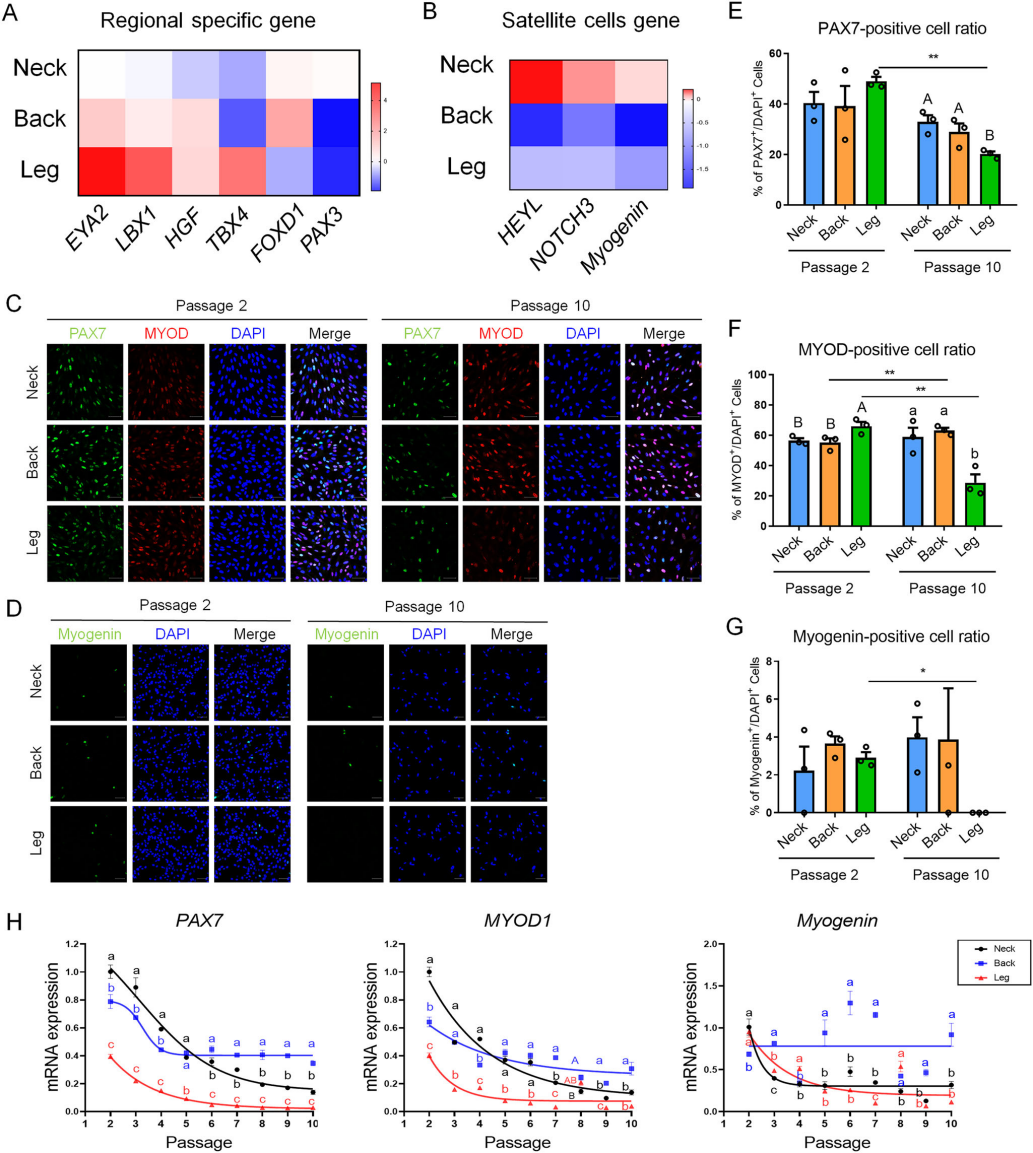
Expression patterns of region-specific and SCs marker genes in PMSCs from different anatomical origins. Heatmap showing the expression of regional-specific genes (**A**) and self-renewal-associated genes (**B**). All heatmap presents DEGs, which are identified with a *p*-value < 0.05 and FC ≥ 3. All heat map expression values were transformed to log_2_ scale (n = 2). **C** Immunofluorescence staining for PAX7 (green, SCs marker) and MYOD (red, myogenic differentiation marker) in PMSCs at passages 2 and 10. DAPI (blue) was used for nuclear staining. **D** Immunofluorescence staining for Myogenin (green) in PMSCs at passages 2 and 10. DAPI (blue) was used for nuclear staining. n = 3; Scale bars: 50 μm. Quantification of PAX7-positive cell ratio (**E**), MYOD-positive cell ratio (**F**), and myogenin-positive cell ratio (**G**) in PMSCs from different regions at passages 2 and passage 10. **H** mRNA expression levels of *PAX7*, *MYOD1*, and *Myogenin* across different passages (P1–P10) in PMSCs derived from the neck (blue), back (black), and leg (red) regions. Gene expression levels decline with increasing passage number, with significant differences among regions (letters indicate statistical significance). The curve for the neck (black), back (blue), and leg (red) were generated using nonlinear regression analysis in GraphPad Prism (n = 3). All data are represented as mean ± SE. Statistically significant differences among the groups were indicated by different letters in Duncan’s multiple range test: lowercase letters (^a–c^) for *p* < 0.01, and uppercase letters (^A–B^) for *p* < 0.05. Student’s *t*-test was employed to assess significant differences between passages 2 and 10 at the same derived region. **p < 0.01 and *p < 0.05.

To investigate regional differences in PMSC characteristics, we analyzed the expression of region-specific genes, SC-associated genes, and myogenic markers in PMSCs derived from the neck, back, and leg regions over multiple passages. RNA-seq analysis revealed that *EYA2*, *LBX1*, and *TBX4* exhibited higher expression levels in leg-derived PMSCs than in the other groups, whereas *FOXD1* showed the highest expression in back-derived PMSCs (Fig. 4A). *PAX3* did not show region-specific expression and showed low expression levels across all three samples, whereas *EYA2* and *HGF* showed the lowest expression levels in neck PMSCs. The self-renewal genes for SCs, *HEYL* and *NOTCH3*, exhibited the highest levels of expression in neck-derived PMSCs (Fig. 4B), indicating different stemness gene signatures in PMSCs.

To further investigate the characteristics of PMSCs according to their site of origin, immunostaining for PAX7, MYOD, and Myogenin was performed (Fig. 4C, D). PAX7 is a well-known marker for SCs^29^. MYOD, which can be co-expressed with PAX7, serves as a marker for activation and early differentiation in PMSCs^30^, whereas Myogenin is recognized as a key marker of muscle cell differentiation^31^. At passage 2, the proportion of PAX7-positive cells was not significantly different among samples; however, by passage 10, PAX7-positive cells significantly decreased in all groups, with leg-derived PMSCs showing the lowest percentage (Fig. 4E). The proportion of MYOD-positive cells was significantly higher in leg-derived PMSCs than in neck- or back-derived PMSCs at passage 2 (Fig. 4F). In addition, leg-derived PMSCs exhibited the lowest MYOD expression levels at passage 10. The MYOD-positive cell ratio of the back-derived PMSCs at passage 10 was higher than that at passage 2 (*p* < 0.01). Contrastingly, the proportion of Myogenin-positive cells was relatively low across all groups, with no significant differences between regions at passage 2. However, at passage 10, Myogenin expression in leg-derived PMSCs was significantly lower than that in the other groups (Fig. 4G).

To further evaluate the myogenic potential of PMSCs over successive passages, we analyzed the mRNA expression levels of *PAX7*, *MYOD1*, and *Myogenin* from passages 2 to 10 (Fig. 4H). Changes in mRNA expression in response to passage progression were assessed using nonlinear regression analysis. The gene expression levels of *PAX7* and *MYOD1* gradually decreased with increasing passage number in all groups. In early passages (approximately until passage 4), neck-derived PMSCs exhibited the highest expression of *PAX7* and *MYOD1*. However, from passage 5 onwards, back-derived PMSCs showed higher expression levels of *PAX7* and *MYOD1*, whereas leg-derived PMSCs consistently exhibited the lowest expression levels across all passages. By contrast, the expression pattern of *Myogenin* differed from those of *PAX7* and *MYOD1*. Neck- and leg-derived PMSCs showed a progressive decline in *Myogenin* expression over successive passages, whereas back-derived PMSCs maintained the highest *Myogenin* expression levels among all three regions.

Thus, these results indicate that PMSCs from different anatomical regions exhibit distinct SC characteristics and myogenic differentiation potentials. Neck-derived PMSCs maintained higher *PAX7* expression, suggesting a greater propensity for self-renewal, whereas back-derived PMSCs exhibited higher *Myogenin* expression, indicating an increased tendency towards differentiation.

### Proliferation capacity of PMSCs from different anatomical regions

**Fig. 5:**
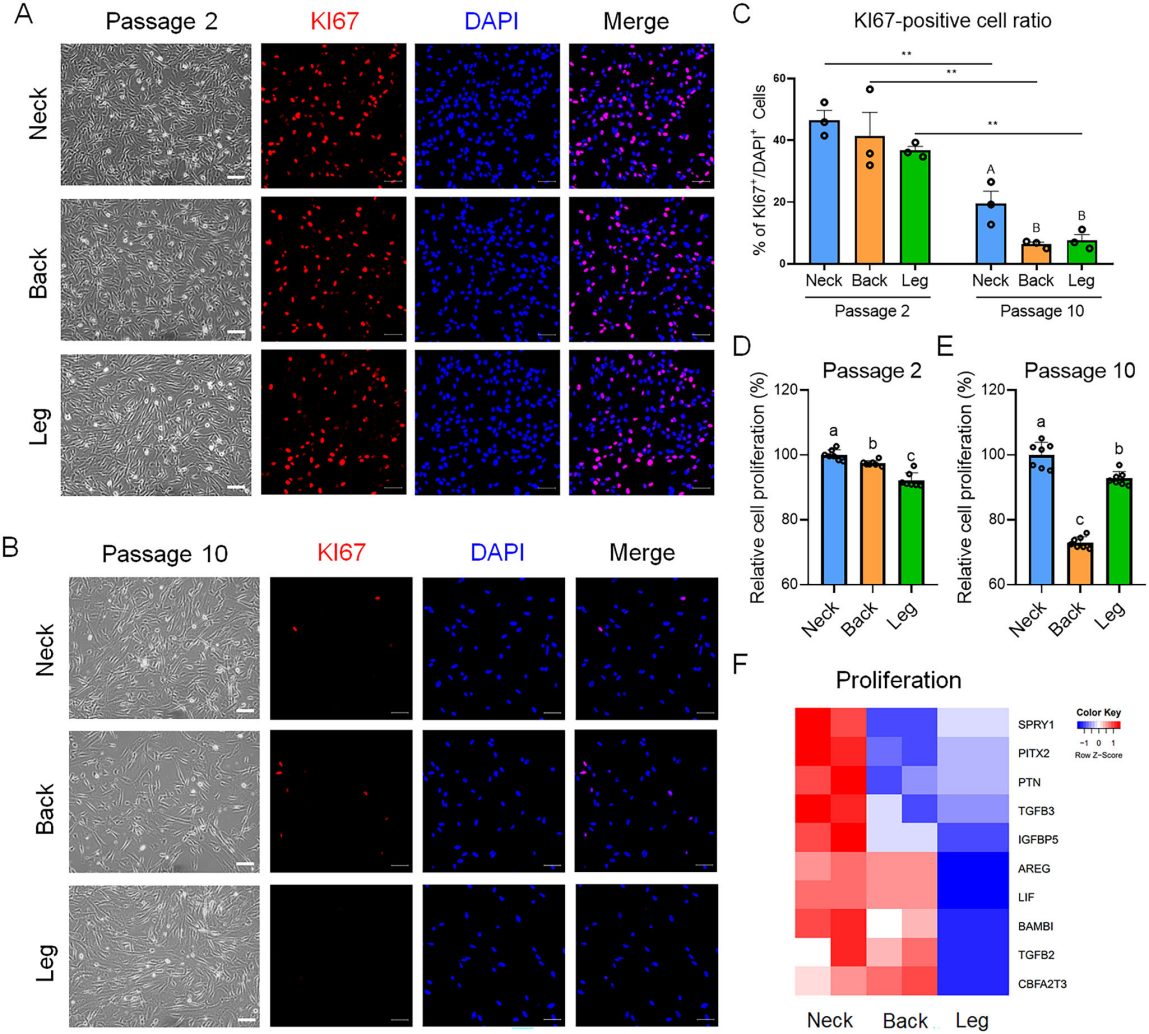
Proliferation capacity of PMSCs derived from different anatomical regions. Morphological and immunocytochemistry images of PMSCs derived from the neck, back, and leg at passage 2 (**A**) and passage 10 (**B**). Cells were stained for KI67 (red) to indicate proliferation and DAPI (blue) to mark nuclei. n = 3; Scale bars: 50 μm. **C** The ratio of KI67^+^/DAPI^+^ cells at passages 2 and 10. **D**, **E** Relative proliferation rate of PMSCs at passage 2 and passage 10. Relative cell proliferation (%) was measured using a cell counting kit (CCK-8; n = 8). **F** Heatmap of proliferation-related genes in PMSCs at passage 2 derived from different anatomical regions. The *p*-value < 0.05 and FC ≥ 3. All data are represented as mean ± SE. Statistically significant differences among the groups were indicated by different letters in Duncan’s multiple range test: lowercase letters (^a–c^) for *p* < 0.01, and uppercase letters (^A–B^) for *p* < 0.05. Student’s *t*-test was employed to assess significant differences between passages 2 and 10 in the same groups. **p < 0.01.

To evaluate the proliferative capacity of PMSCs derived from different anatomical regions, we cultured cells up to passage 10 for 30 days. At passage 2, all groups retained a spindle-like morphology, whereas at passage 10, an increase in cell length was observed across all regional groups (Fig. 5A, B). To assess cell proliferation, the cells were immunostained for KI67, a well-known proliferation marker, at passages 2 and 10 (Fig. 5A, B). At passage 2, all three groups exhibited high KI67 expression, indicating a robust proliferative capacity, with no significant differences among them (Fig. 5C). However, at passage 10, KI67 expression significantly decreased across all groups, with neck-derived PMSCs maintaining the highest proliferation rate, whereas back-derived PMSCs exhibited the lowest rate (Fig. 5C).

For a more precise quantification of proliferation, the CCK-8 assay was performed (Fig. 5D, E). Because CCK-8 measurements were performed separately for each passage, the proliferation of back- and leg-derived PMSCs was expressed relative to the neck-derived PMSCs within the same passage (set as 100%). At passage 2, neck-derived PMSCs showed the highest proliferation rate, followed by back- and leg-derived PMSCs (Fig. 5D). Although KI67 expression significantly decreased across all groups at passage 10 (Fig. 5C), neck-derived PMSCs showed a higher proliferation rate, whereas back-derived PMSCs exhibited the lowest proliferation capacity (Fig. 5E).

To further investigate the molecular basis of these differences in proliferation, we performed a heatmap analysis of the proliferation-related gene expression (Fig. 5F). Genes associated with cell proliferation, including *SPRY1*, *PITX2*, *PTN*, *TGFB3*, and *IGFBP5*, were significantly upregulated in neck-derived PMSCs compared to those in back- and leg-derived PMSCs. By contrast, leg-derived PMSCs showed lower expression of proliferation-related genes, such as *AREG*, *LIF*, *BAMBI*, *TGFB2*, and *CBFA2T3*, indicating their reduced proliferative potential.0 Thus, these findings suggest that neck-derived PMSCs possess the highest proliferative capacity, making them an optimal cell source for large-scale expansion, whereas back- and leg-derived PMSCs exhibit lower proliferative potential, which may limit their application in large-scale expansion.

### Regional differences in myogenic differentiation and fiber type composition

**Fig. 6:**
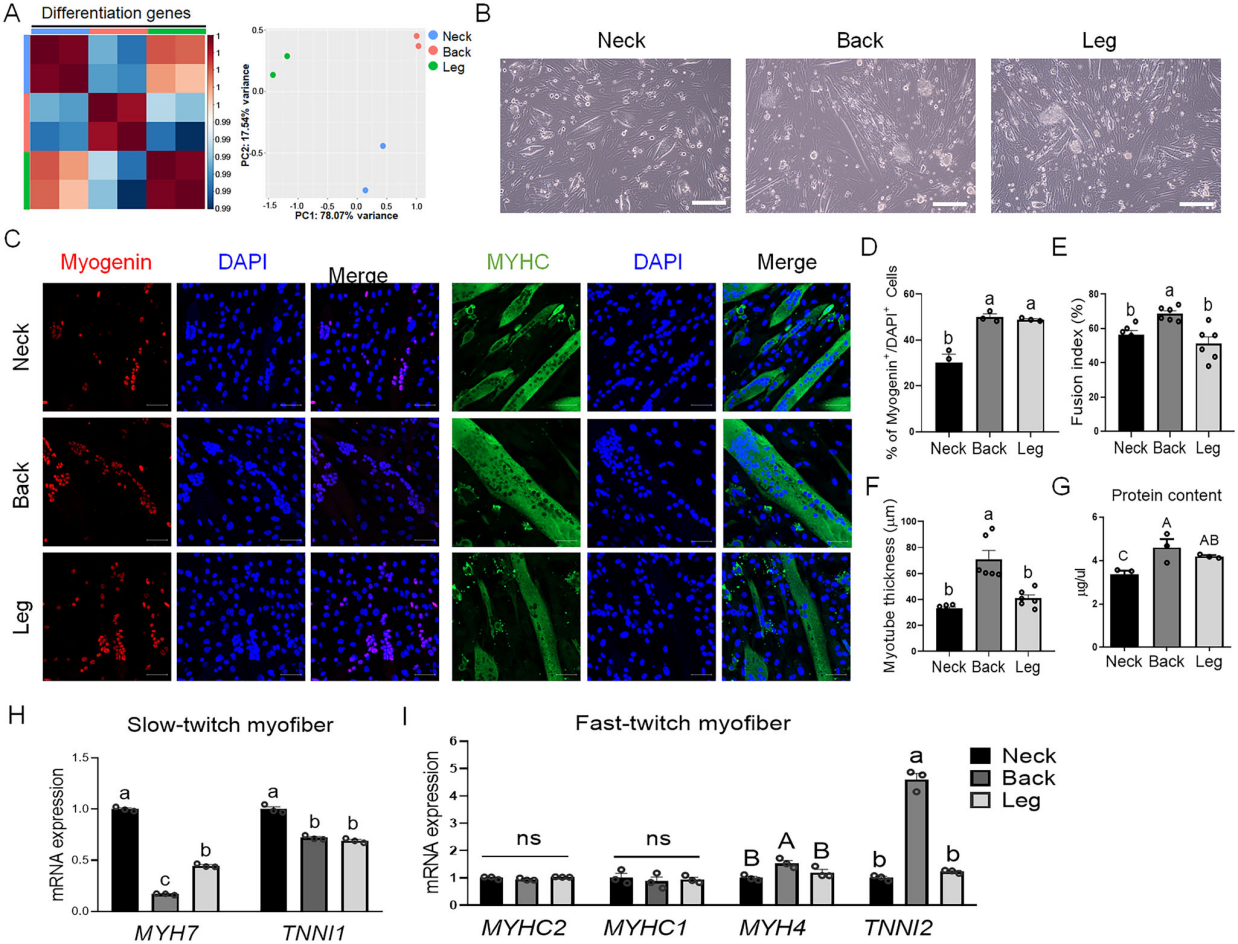
Comparative analysis of myogenic differentiation and fiber type composition among neck, back, and leg muscle-derived cells. **A** Correlation score matrix (left) and principal component analysis (PCA, right) of differentiation-related gene expression in cells differentiated from PMSCs derived from the neck, back, and leg. **B** Representative bright-field images of PMSCs from each region on day 5 after differentiation. n = 3; Scale bars: 200 μm. **C** Immunofluorescence staining for myogenin (red) and MYHC (green) in myotubes differentiated from PMSCs. n = 6; Scale bars: 50 μm. **D** Quantification of myogenin⁺/DAPI⁺ cells in myogenic cultures from different anatomical regions. Different letters indicate significant differences (*p* < 0.05). **E** Myotube fusion index calculated based on multinucleated myotubes (*p* < 0.05). **F** Measurement of myotube thickness in differentiated cultures. **G** Total protein content of differentiated PMSCs. **H** Comparison of expression levels of slow-twitch muscle fiber-related genes (*MYH4* and *TNNI1*) among the three groups. **I** Comparison of expression levels of fast-twitch muscle fiber-related genes (*MYH2*, *MYHC1*, *MYH4*, and *TNNI2*) among the three groups. All data are represented as mean ± SE. Statistically significant differences among the groups were indicated by different letters in Duncan’s multiple range test: lowercase letters (^a–c^) for *p* < 0.01, and uppercase letters (^A–B^) for *p* < 0.05.

To investigate the myogenic differentiation potential of PMSCs, we first evaluated whether the expression of differentiation-related genes in PMSCs was distinct before the induction of differentiation (Fig. 6A). The expression patterns of genes associated with myogenic differentiation varied among the groups, suggesting that the differentiation characteristics of PMSCs differ depending on their anatomical region. Next, to evaluate differentiation potential and myotube maturation, PMSCs were induced to differentiate for 5 days (Fig. 6B), and quantitative analysis of Myogenin and MYHC by immunostaining was caried out (Fig. 6C). Morphological differences observed by bright-field imaging was not evident, but immunofluorescence staining for myogenin and MYHC showed a significantly lower proportion of Myogenin⁺/DAPI⁺ cells in neck-derived PMSCs compared to those from the back and leg (Fig. 6C, D). The fusion index was significantly higher in back-derived cells than that in the neck- or leg-derived cells (Fig. 6E). Moreover, the myotube thickness was greater in back-derived cultures, whereas neck- and leg-derived cells formed significantly thinner myotubes (Fig. 6F), indicating enhanced myotube differentiation and maturation in back-derived PMSCs. Furthermore, the protein contents were significantly different among the groups (Fig. 6G). The back-derived PMSCs exhibited the highest protein content, whereas the neck-derived PMSCs showed the lowest protein content.

To further investigate the molecular basis of regional differences in differentiated myotube types, we analyzed the expression of slow- and fast-twitch myofiber-related genes across the groups. The expression levels of *MYH7* (encoding slow-twitch myosin heavy chain) and *TNNI1* (a slow-twitch troponin marker) were significantly higher in the neck-derived cells than in the back- and leg-derived cells (Fig. 6H). By contrast, the expression of fast-twitch fiber markers, including *MYH4* and *TNNI2*, was significantly upregulated in back-derived PMSCs compared to that in neck- and leg-derived cells, indicating a higher propensity for fast-twitch muscle differentiation, although there was no significant difference in *MYHC2* and *MYHC1* expression among the groups (Fig. 6I). Together, these findings suggest that back-derived PMSCs exhibit enhanced myotube differentiation potential and that neck-derived cells favor slow-twitch muscle fiber characteristics, whereas back-derived cells preferentially differentiate into fast-twitch fibers.

## Discussion

In this study, we compared PMSCs from three different regions, including the neck, back, and leg, and found that each PMSC group exhibited distinct characteristics such as global gene expression patterns, proliferation rate, *HOX* gene expression patterns, differentiation abilities, myotube formation, and fiber type composition. These findings provide valuable insights for selecting the appropriate anatomical region for PMSC isolation, as the distinct characteristics of PMSCs from different sources may influence their suitability for specific experiments or applications, such as cultured meat production. SCs sourced from diverse anatomical regions, rather than restricted to specific sites, are currently used for cultured meat production. However, our findings revealed that neck-derived PMSCs exhibited superior proliferative capacity, whereas back-derived PMSCs maintained high expression of the SC markers, *PAX7* and *MYOD*, during long-term culture. In addition, back-derived PMSCs demonstrated an increased efficiency in myotube formation. Therefore, an appropriate combination of these two cell sources can be used to develop an optimized cultured meat production strategy for pigs.

The number of SCs present in the muscles varies depending on the muscle region and myofiber type^5,7,9^. In particular, the muscles containing slow-twitch fibers have been observed to have a greater concentration of SCs than those containing fast-twitch fibers^5–7,32^. Regarding proliferation, SCs residing in slow-twitch myofibers exhibit a higher proliferation capacity compared to those in fast-twitch myofibers in mice^33–35^. This was also consistent with our results, which showed that the cell extraction yield and proliferation rate were highest in neck-derived PMSCs, which contained a higher proportion of slow-twitch myofibers than back- and leg-derived PMSCs. However, muscle fiber composition can vary based on factors such as breed and age, which necessitates caution when interpreting these results. To achieve a more precise understanding, further studies are needed to investigate myofiber composition in neonatal pigs.

We demonstrated that neck-derived PMSCs preferentially differentiated into slow-twitch muscle fibers, whereas back-derived PMSCs favored fast-twitch fiber differentiation. Typically, the proportion of slow- and fast-twitch fibers varies across muscle regions, making it unclear whether SCs derived from specific muscle regions consistently differentiate into a single fiber type. The longissimus muscle, one of the muscles in the back, contains a significant amount of fast-twitch myofibers, which are designed for quick, powerful movements^36^. The *semispinalis capitis* from the neck is a muscle involved in maintaining posture, with a higher proportion of slow-twitch myofibers than the back and leg muscles^37^. In differentiation experiments conducted using highly isolated site-specific PMSCs, the cells tended to differentiate into muscle types that resembled those of their original anatomical origin. These findings suggest that PMSCs retain differentiation characteristics reflective of their muscle region of origin even under controlled in vitro conditions. Additionally, their anatomical origin may influence their tendency to differentiate toward a higher proportion of either fast- or slow-twitch fibers.

The histochemical characteristics of muscle, including fiber type composition, significantly influence the energy metabolism patterns of muscles and subsequently impact meat, such as texture and flavor, which can also affect consumer preferences^38^. Positive evaluations of muscle fiber softness may be related to the oxidative capacity and fat content of the muscle fibers^38,39^. Slow-twitch myofibers primarily utilize oxidative metabolism and store neutral lipids as their main energy source, whereas fast-twitch myofiber type II mainly relies on anaerobic glycolysis and stores glycogen with relatively low neutral lipid content^40,41^. Muscles with a higher proportion of oxidative fibers and greater lipid content generally exhibit superior tenderness^39^. We identified distinct differences in the types of myofibers generated from PMSCs derived from anatomically distinct muscles. Furthermore, we showed that the protein content varied among the PMSCs derived from the neck, back, and legs. The consumers are progressively favoring health foods characterized by high-protein and low-fat content^42,43^. Given the increasing demand for high-protein meats, cultured meat with enhanced protein content may align with consumer health preferences and increase market acceptance. Therefore, the selection of muscle cell sources that can improve the nutrient profile is important in commercializing cultured meat. This study emphasizes the cell source selection strategies and suggests that the differentiation characteristics and protein content of muscle cells, depending on their anatomical origin, may partially influence the taste, nutritional value, and consumer acceptance of the final cultured meat products.

The expression of region-specific genes, particularly *HOX* genes, has been documented in the skeletal muscle of adult mice, adult pigs, and newborn calves, showing notable differences among different muscle types^45–48^. Consistent with previous findings, our study revealed that region-specific *HOX* gene expression profiles reflect an embryonic origin. *HOX* genes associated with anterior development are highly expressed in neck-derived PMSCs, whereas those involved in posterior development are predominantly expressed in leg-derived PMSCs. Thus, this suggests that SCs cultured under identical conditions retain the gene expression patterns related to embryonic development in each region.

While *HOX* genes are traditionally recognized for their homeotic functions, they also play non-homeotic roles in regulating other cellular functions, such as myogenic regulatory factors (MRFs)^44,49–51^. For example, HOXA11, an upstream regulator of *MYOD*, is highly expressed in muscle cells derived from the *semimembranosus* (SM) of the leg, where the differentiation rate is lower than that of muscle cells derived from the back in Hanwoo^44^. This finding is consistent with our results, which showed a low differentiation tendency in leg-derived PMSCs with elevated *HOXA11* expression. Additionally, the Hoxa9 inhibits the Wnt, TGFβ, and JAK/STAT signaling pathways, leading to the induction of senescence in young mouse SCs^52,53^. Moreover, the deletion of *Hoxa9* in aged mouse SCs enhanced their regenerative capacity. In this study, we found that leg-derived PMSCs with high *HOXA9* expression exhibited the fastest decline in stemness genes *PAX7* and *MYOD1* during long-term culture (Fig. 4H). Furthermore, leg-derived PMSCs showed the lowest proliferation among the three regions, with reduced expression of genes related to positive cell division and the TGFβ signaling pathway, which is critical for cell proliferation. These findings suggest that differences in *HOX* gene expression may influence the distinct characteristics of SCs derived from different anatomical regions, potentially affecting their regenerative abilities and behaviors^50,52^.

The fate of multipotent progenitor cells is primarily influenced by their spatial positioning and interactions with adjacent tissues such as the neural tube, notochord, myotome, and dorsal ectoderm^9,15,16^. Differences in gene expression involved in regional specification persist into adulthood^23^, although the expression of some specific genes may decrease or cease during growth^54–56^. EYA, in collaboration with SIX1 and SIX4, function as co-activators of Myf5 and MyoD in the limb and hypaxial somites^57–59^. Ono et al^35^. further demonstrated that *Eya2* is highly expressed in slow-dividing adult mouse SCs, which possess a long-term self-renewal capacity. Contrary to these findings, our results suggest that leg-derived PMSCs, despite exhibiting high *EYA2* expression and slow proliferation, showed significantly lower levels of the self-renewal-associated genes *HEYL* and *NOTCH3* than PMSCs from other regions. This suggests that while the upregulation of *EYA2* is associated with slow proliferation, it may not be sufficient to maintain long-term self-renewal in certain SC populations, such as those from the leg, where other regulatory factors might play a more critical role.

In this study, *LBX1 and TBX4* were highly expressed exclusively in leg-derived PMSCs but not in neck- and back-derived PMSCs. *TBX4* plays a critical role in hind limb development, and in embryos, its loss leads to severe hindlimb developmental defects^60,61^. *TBX4* expression in postnatal leg muscle cells is higher than that in stem cells from back muscle cells^44^. Additionally, Chao et al^62^. demonstrated that *Lbx1* was highly expressed in porcine limbs, semitendinosus, and longissimus dorsi muscle tissues. *FOXD1* was highly expressed in back-derived PMSCs, but not in neck- and leg-derived PMSCs. Walker et al^63^. reported that *Foxd1* expression was confined to cells in the dorsal dermis among precursor-derived fibroblasts. Although additional research is required to elucidate the role of *FOXD1* in SCs, our study demonstrated that its elevated expression may be a distinguishing characteristic of back-derived PMSCs. *PAX3* did not exhibit region-specific expression and showed low expression levels across all three samples. PAX3 functions in both trunk and limb muscle precursor cells, but is not essential for limb muscle development^20,64^. In addition, Pax3 is highly expressed in specific regional muscles, such as trunk and diaphragm muscles, compared to limb-derived muscle cells^9,65^. The precise functions of *LBX1*, *HGF*, *TBX4*, *FOXD1*, and *PAX3* within SCs are yet to be fully elucidated. However, differences in the expression patterns of these genes across muscle regions suggest that they may contribute to the unique characteristics of SCs in distinct anatomical locations. This supports the notion that SCs exhibit heterogeneity in their proliferation and regenerative capacities, depending on the anatomical position of the muscle^12,52^.

Myogenesis in SCs is stringently regulated by MRFs, which play crucial roles in orchestrating proliferation and differentiation necessary for efficient muscle development and regeneration^5,9^. Consequently, these myogenic transcription factors serve as key molecular markers of SC states and guide processes such as quiescence, activation, differentiation, and the eventual loss of regenerative potential. Maintenance of PAX7 expression is important for preserving the stemness of SCs in long-term culture^66^. In our experiments, neck-derived PMSCs expressed higher levels of myogenic transcription factors and self-renewal genes at early passages than back- or leg-derived PMSCs. However, from passage 5 onwards, myogenic transcription factors (*PAX7*, *MYOD1*, and *Myogenin*) were consistently expressed at higher levels in back-derived PMSCs. By contrast, leg-derived PMSCs exhibited lower levels of self-renewal markers, such as *HEYL* and *NOTCH3*, along with consistently low expression of PAX7 and MYOD from passages 2 to 10. This suggests that leg-derived PMSCs lose their stemness most rapidly among the three groups during long-term culture, as evidenced by the consistently low expression of key myogenic and self-renewal markers over multiple passages. Thus, neck-derived PMSCs are better suited for short-term expansion, whereas back-derived PMSCs have a potential advantage in long-term culture.

This study used PMSCs isolated from neonatal pigs, which typically contain a higher proportion of satellite cells and exhibit robust proliferative and regenerative potential^67^. However, PMSCs derived from adult animals may show different regional characteristics or altered cellular behaviors, and thus, additional validation using adult-derived cells will be necessary. Furthermore, RNA-seq analyses were performed with two biological replicates due to limited cell availability, indicating that future studies with larger replicate numbers will be needed to strengthen these findings.

In conclusion, this study suggests that the anatomical origin of PMSCs has a substantial impact on their gene expression profiles, proliferation, differentiation, and myotube-forming capacity. Our findings highlighted the importance of selecting an appropriate cell extraction site, a factor that remains relatively underexplored in cultured meat production. Based on a comprehensive evaluation of their proliferation capacity in both short- and long-term cultures as well as their myogenic differentiation and myotube formation abilities, we conclude that while neck-, back-, and leg-derived PMSCs exhibit distinct characteristics and excel in specific aspects, back-derived PMSCs offer the most balanced proliferation and differentiation potential, making them the most promising candidate for porcine cultured meat production. Furthermore, differences in PMSCs origin may not only affect cell differences characteristics but could also impact quality attributes of cultured meat, such as texture, nutritional composition, and flavor.

## Methods

### Animals and PMSCs isolation

All animal experiments were conducted according to the guidelines and regulations of Jeonbuk National University, compliant with the ARRIVE guidelines, and approved by the Animal Ethics Committee of Jeonbuk National University (Permit No. JBNU, NON2023-037-003).

In this study, we used three 1-day-old male LYD (Landrace × Yorkshire × Duroc). PMSCs were isolated as described previously^68^. Pigs were euthanized using Zoletil anesthesia and CO_2_ inhalation, depending on experimental requirements. For CO_2_ euthanasia, the pigs were euthanized by gradual CO₂ inhalation at 50% chamber volume per minute to a final concentration of ≥80%. Briefly, we isolated PMSCs from the neck (*Semispinalis capitis*), back (*Longissimus dorsi*), and leg (*Biceps femoris*) tissues. Approximately 2 g of neck muscle, 5.7 g of back muscle, and 4.5 g of leg muscle tissues were collected for PMSC isolation. Tissues collected from each muscle region were washed with phosphate-buffered saline (PBS; Gibco, USA) containing 10% penicillin–streptomycin (PS; Gibco) and minced for 5 min. The shredded muscle tissue was dissociated into the following digestion medium: 2 mg/mL Collagenase D (Roche), 1 U/mL dispase II (Roche, Germany), 0.25% trypsin-EDTA (Gibco), and 10% PS in Dulbecco’s modified Eagle medium/nutrient mixture F-12 (DMEM/F12; Gibco). The cell slurries were neutralized using DMEM/F12 supplemented with 10% fetal bovine serum (FBS; Gibco) and 1% penicillin, streptomycin glutamine (PSG; Gibco). The neutralized solution was sequentially filtered through 100 and 70 μm cell strainers, then centrifuged at 204 × *g* for 5 min. Ammonium chloride potassium lysing buffer (Gibco) was used to remove red blood cells according to the manufacturer’s guidelines. The cells were resuspended in DMEM/F12 supplemented with 15% FBS, 1% PSG, and 5 ng/mL basic fibroblast growth factor (bFGF; Gibco) and seeded in 0.15% gelatin-coated dishes.

The cells were cultured for 72 h and then detached using trypsin-EDTA (Gibco) to purify the PMSCs. The cells were reconstituted using fluorescence-activated cell sorting (FACS) solution (1% bovine serum albumin [BSA] in PBS) and stained with APC anti-human CD29 antibody (1:20; BioLegend, USA), PE-Cy^TM7^ anti-human CD56 antibody (1:20; BD Biosciences, USA), FITC anti-sheep CD31 (1:20; Bio-Rad, USA), and FITC anti-sheep CD45 (1:20; Bio-Rad) for 30 min on ice. Following antibody incubation, the cells were washed twice with chilled FACS solution and resuspended in DMEM/F12 supplemented with 15% FBS. Cell sorting was performed using a FACS Aria III flow cytometer (BD Biosciences). The CD31^−^/CD45^−^/CD29^+^/CD56^+^ cells were identified as SCs.

### Cell culture and morphological analysis

PMSCs were cultured in DMEM/F12 supplemented with 15% FBS, 1% PSG, and 5 ng/mL of bFGF in a humidified incubator at 37 °C with 5% CO_2_. The medium was replaced every alternate day to support cell proliferation. For differentiation, PMSCs from each anatomical region at passage 3 were used. The proliferation media was replaced to DMEM/high glucose (Hyclone, USA) supplemented with 5% horse serum, 1% PSG, and 1 μg/mL of insulin solution (Sigma Aldrich, USA). The differentiation medium was changed every 2 days. The morphology was observed using an inverted microscope.

### Sample and library preparations

Total RNA was extracted from the neck, back, and leg PMSCs at passage 2 using TRIzol reagent (Invitrogen, USA), according to the manufacturer’s instructions. RNA quality was evaluated using an Agilent 2100 Bioanalyzer with an RNA NanoChip (Agilent Technologies, Ireland), following the manufacturer’s guidelines. RNA was quantified using the Quant-iT RiboGreen RNA Kit (Invitrogen). The mRNA libraries were produced using the TruSeq Stranded mRNA Sample Preparation Kit (Illumina) according to the manufacturer’s protocols. Briefly, poly A-containing RNA was purified using poly T oligo-attached magnetic beads and fragmented into small sections using divalent cations. The fragmented RNAs were used to synthesize first-strand cDNA using reverse transcriptase and random primers. Actinomycin D was added to prevent non-specific DNA-dependent synthesis. The RNA template was removed, and dTTP (deoxythymidine triphosphate) was replaced with dUTP (deoxyuridine triphosphate) in the synthesized replacement strand to generate double-stranded cDNA. A single adenine nucleotide was then attached to the 3′ end of the blunt-ended fragment. Subsequently, the adapter was ligated to the ends of the double-stranded cDNA fragments, and PCR was used to amplify the DNA in the library. Library quality was analyzed using the Bioanalyzer DNA 1000 Chip (Agilent Technologies). RNA sequencing was performed using an Illumina NextSeq 500 system by the LAS company (Gimpo, Republic of Korea).

### RNA-seq data analysis

Read alignment was performed against a porcine reference genome assembly (susScr 11) using STAR (v2.7.9a)^69^. Mapped reads were quantified using VERSE (v0.1.5)^70^ and normalized using the median of ratios method in DESeq2 (v1.42.1)^71^. For data visualization, a correlation score matrix was generated using the corrplot package (v0.94) in R^72^. A principal component analysis (PCA) plot was produced using the ggplot2 (v3.5.1)^73^ package in R, and a heatmap was generated using the heatmap.2 function of the gplots package (v3.2.0) in R^74^. Differentially expressed genes (DEGs) were identified based on fold change (FC) ≥ 3 and *p*-value < 0.05, with clustering performed using the hclust function in R^74^. Gene ontology (GO) analysis was performed using the DAVID web tool^75^. GO term results were visualized using ggplot2 (bubble or bar plot). The scatter plot was generated using the base plot function in R, with gene expression data classified by FC ≥ 3, *p*-value < 0.05, and normalized fragment level ≥2.

### Cell viability assay

Cells were seeded in a 0.15% gelatin-coated 96-well plate (5 × 10^3^) and cultured in DMEM/F12 containing 15% FBS, 1% PSG, and 5 ng/mL bFGF. After 72 h of culture, the cells were incubated with cell counting kit-8 (CCK-8; Dojindo, Japan) for 3 h. The viability of cells derived from three different parts was analyzed, with each part tested in seven replicates. Absorbance was measured at 450 nm using a microplate reader (Thermo Fisher Scientific).

### Immunofluorescent analysis

Cultured cells were fixed using 4% paraformaldehyde in PBS for 20 min at 4 °C and washed three times with PBS. The fixed cells were blocked with 3% BSA and 0.3% Triton X-100 in PBS for 2 h at room temperature. After washing three times with washing solution (0.3% Triton X-100 in PBS), the cells were incubated overnight at 4 °C with the following primary antibodies: anti-KI67 (1:100; GeneTex, USA), anti-PAX7 (1:20; DSHB, USA), anti-MYOD (1:200; Proteintech, USA), anti-Myogenin (1:50; DSHB), and anti-MYHC (1:40; DSHB). The cells were washed thrice with a washing solution. The cells were then incubated for 2 h at room temperature with fluorescent secondary antibodies Alexa Fluor 488 or 568 (1:1000; Abcam, UK). Secondary antibodies corresponding to primary antibodies were used. The nuclei were visualized by staining with 4′-6-diamidino-2-phenylinodole (DAPI) for 5 min at room temperature, and the cells were washed with washing solution three times. Fluorescent images were obtained using a super-resolution confocal laser scanning microscope (SR-CLSM; LSM 880, Carl Zeiss, Germany) and processed using the Zen 2.6 software (Carl Zeiss). Three random fields were acquired to manually count the nuclei individually labeled with KI67-, PAX7-, MYOD-, and Myogenin-positive nuclei. MYHC fluorescence images were obtained from six random fields to measure fusion index and myotube thickness. The fusion index was calculated as the ratio of the total number of nuclei within the myotubes containing two or more nuclei to the total number of nuclei in the MYHC images. Myotube thickness was manually quantified using ImageJ software.

### Protein contents

The total protein contents were analyzed using the Bradford method. Briefly, the differentiated muscle cells were kept on ice for 40 min in 200 µl of radioimmunoprecipitation assay buffer (Biosesang, Korea) that include protease inhibitors (Thermo Fisher Scientific) with vortexing. The samples were then centrifuged at 21,000 × *g* for 30 min. The supernatant was collected, and the protein concentration was measured using the detergent-compatible protein assay kit (Bio-Rad, USA).

### RNA extraction and RT qPCR

RNA was isolated using the RNeasy Mini kit (Qiagen, Germany), and 1 μg of RNA was used to synthesize cDNA using SuperScript Ⅲ reverse transcriptase (Invitrogen, USA) and poly T oligonucleotides (Invitrogen), and 10 mM dNTP Mix (Invitrogen) following the manufacturer’s guidelines. RT-qPCR was conducted using TOPrealtm qPCR 2ⅹ premix (Enzynomics, Korea). The thermal cycling protocol of PCR consisted of 40 cycles at 95 °C for 10 s, 60 °C for 15 s, and 72 °C for 20 s. The primer sequences are listed in Table 2.

**Table 2:** Primer sequences used in RT-qPCR

Gene name	Primer sequences	Accession number	Length (bp)
*GAPDH*	F: ACCCAGAAGACTGTGGATGG R: AAGCAGGGATGATGTTCTGG	NM_001206359.1	79
*HOXA2*	F: ACTGCTTACACCAACACGCA R: TTGTGCTTCATCCTCCGGTT	XM_003134843.5	155
*HOXC8*	F: TGGTGCAATATCCCGACTGT R: AGACTTCAATCCGGCGCTTT	XM_021091732.1	225
*HOXA10*	F: TGGAGCTGGAGAAGGAGTTT R: GTTTCATCCTGCGGTTCTGA	XM_005673295.3	126
*HOXA11*	F: ATTGAGCCCGCCACTAAATG R: TGCCCACGGTGCTATAGAAA	XM_003134850.4	196
*HOXC11*	F: AGCGCTGCCCTTATTCGAAA R: AAATACTGCAGCCGGTCTCT	XM_021091724.1	190
*HOXA13*	F: GCCAAATGTACTGCCCCAAA R: AGCGTATTCCCGTTCGAGTT	NM_001434950.1	167
*PAX7*	F: TCCAGCTACTCCGACAGCTT R: TGCTCAGAATGCTCATCACC	XM_021095458.1	100
*MYF5*	F: AGACGCCTCAAGAAGGTCAA R: AGCCTCTGGTTGGGGTTAGT	NM_001278775.1	74
*MYOD*	F: GTGCAAACGCAAGACCACTA R: GCTGATTCGGGTTGCTAGAC	NM_001002824.1	128
*Myogenin*	F: CCACTTCTATGACGGGGAAA R: GGTCCACAGACACGGACTTC	NM_001012406.1	203
*MYHC1*	F: AGGAAAGTCGCAGAACAGGA R: TCCATCTCTCCCTGGATTTG	NM_001104951.2	131
*MYHC2*	F: CCCTGAATGACACAGTGGTG R: CAGTTTGAGCCCCAGAGAAG	NM_214136.1	81
*MYH4*	F: AGAGAGGAGCAGGAGAGTGG R: TGTCCTCCATCTCTCCCTGG	NM_001123141.1	137
*MYH7*	F: ATGAAGGAGGAGTTTGGGCG R: AGCTGCAGGTCGTTCTTCTC	NM_213855.2	113
*TNNI1*	F: CCTGCTGGGCTCTAAACACA R: ACATGGCCTCGACGTTCTTT	NM_213912.3	112
*TNNI2*	F: CTGAGAAGGGTGCGGATGTC R: CCGTGTCCTCCTTCTTGACC	NM_001032359.1	102

### Statistical analysis

All statistical analyses were conducted using the SAS software (v. 9.4; SAS Institute Inc., Cary, NC, USA). To compare the statistical differences, we used Student’s *t*-test or one-way analysis of variance (ANOVA) followed by Duncan’s Multiple Range Test to compare the differences of anatomical PMSCs. All data are presented as mean ± standard error (SE), with statistical significance at *p* < 0.01 and *p* < 0.05.

## Supplementary information

Supplementary materials_pdf

## Data Availability

Relevant data are available on request from the corresponding author upon reasonable request.

## References

[1] Datar, I. & Betti, M. Possibilities for an in vitro meat production system. *Innov. Food Sci. Emerg. Technol.* **11**, 13–22 (2010).

[2] Hong, T. K., Shin, D.-M., Choi, J., Do, J. T. & Han, S. G. Current issues and technical advances in cultured meat production: a review. *Food Sci. Anim. Resour.* **41**, 355 (2021). 34017947 10.5851/kosfa.2021.e14 PMC8112310

[3] Post, M. J. Cultured beef: medical technology to produce food. *J. Sci. Food Agric.* **94**, 1039–1041 (2014). 24214798 10.1002/jsfa.6474

[4] Post, M. J. et al. *Sci. Sustain. Regul. Chall. Cult. Meat* **1**, 403–415 (2020).

[5] Collins, C. A. et al. Stem cell function, self-renewal, and behavioral heterogeneity of cells from the adult muscle satellite cell niche. *Cell* **122**, 289–301 (2005). 16051152 10.1016/j.cell.2005.05.010

[6] Ding, S. et al. Characterization and isolation of highly purified porcine satellite cells. *Cell Death Discov.* **3**, 1–11 (2017). 10.1038/cddiscovery.2017.3 PMC5385392 28417015

[7] Keefe, A. C. et al. Muscle stem cells contribute to myofibres in sedentary adult mice. *Nat. Commun.* **6**, 7087 (2015). 25971691 10.1038/ncomms8087 PMC4435732

[8] Redshaw, Z., McOrist, S. & Loughna, P. Muscle origin of porcine satellite cells affects in vitro differentiation potential. *Cell Biochem. Funct.* **28**, 403–411 (2010). 20589736 10.1002/cbf.1670

[9] Yin, H., Price, F. & Rudnicki, M. A. Satellite cells and the muscle stem cell niche. *Physiol. Rev.* **93**, 23–67 (2013). 23303905 10.1152/physrev.00043.2011 PMC4073943

[10] Biressi, S. & Rando, T. A. Heterogeneity in the muscle satellite cell population. *Semin. Cell Dev. Biol.* **21***,* 845–854 (2010). 10.1016/j.semcdb.2010.09.003 PMC2967620 20849971

[11] Kalhovde, J. et al. Fast’and ‘slow’muscle fibres in hindlimb muscles of adult rats regenerate from intrinsically different satellite cells. *J. Physiol.* **562**, 847–857 (2005). 15564285 10.1113/jphysiol.2004.073684 PMC1665547

[12] Ono, Y., Boldrin, L., Knopp, P., Morgan, J. E. & Zammit, P. S. Muscle satellite cells are a functionally heterogeneous population in both somite-derived and branchiomeric muscles. *Dev. Biol.* **337**, 29–41 (2010). 19835858 10.1016/j.ydbio.2009.10.005 PMC2806517

[13] Pallafacchina, G., Blaauw, B. & Schiaffino, S. Role of satellite cells in muscle growth and maintenance of muscle mass. *Nutr. Metab. Cardiovasc. Dis.* **23**, S12–S18 (2013). 22621743 10.1016/j.numecd.2012.02.002

[14] Komiya, Y. et al. Correlation between skeletal muscle fiber type and responses of a taste sensing system in various beef samples. *Anim. Sci. J.* **91**, e13425 (2020). 32691493 10.1111/asj.13425 PMC7507124

[15] Mootoosamy, R. C. Distinct regulatory cascades for head and trunk myogenesis. *Development* **129**, 573–83 (2002). 10.1242/dev.129.3.573 11830559

[16] Porter, J. D. et al. Distinctive morphological and gene/protein expression signatures during myogenesis in novel cell lines from extraocular and hindlimb muscle. *Physiol. Genom.* **24**, 264–275 (2006). 10.1152/physiolgenomics.00234.2004 16291736

[17] Heude, E. et al. Unique morphogenetic signatures define mammalian neck muscles and associated connective tissues. *Elife* **7**, e40179 (2018). 30451684 10.7554/eLife.40179 PMC6310459

[18] Yahya, I., Morosan-Puopolo, G. & Brand-Saberi, B. The CXCR4/SDF-1 Axis in the development of facial expression and non-somitic neck muscles. *Front. Cell Dev. Biol.* **8**, 615264 (2020). 33415110 10.3389/fcell.2020.615264 PMC7783292

[19] Noden, D. M. & Francis-West, P. The differentiation and morphogenesis of craniofacial muscles. *Dev. Dyn. Off. Publ. Am. Assoc. Anat.* **235**, 1194–1218 (2006). 10.1002/dvdy.20697 16502415

[20] Braun, T. & Gautel, M. Transcriptional mechanisms regulating skeletal muscle differentiation, growth and homeostasis. *Nat. Rev. Mol. Cell Biol.* **12**, 349–361 (2011). 21602905 10.1038/nrm3118

[21] Dietrich, S. Regulation of hypaxial muscle development. *Cell Tissue Res.* **296**, 175–182 (1999). 10199977 10.1007/s004410051278

[22] Buckingham, M. Myogenic progenitor cells and skeletal myogenesis in vertebrates. *Curr. Opin. Genet. Dev.* **16**, 525–532 (2006). 16930987 10.1016/j.gde.2006.08.008

[23] Harel, I. et al. Distinct origins and genetic programs of head muscle satellite cells. *Dev. Cell* **16**, 822–832 (2009). 19531353 10.1016/j.devcel.2009.05.007 PMC3684422

[24] Apiou, F. et al. Fine mapping of human HOX gene clusters. *Cytogenet. Genome Res.* **73**, 114–115 (1996). 10.1159/000134320 8646877

[25] Garcia-Fernàndez, J. The genesis and evolution of homeobox gene clusters. *Nat. Rev. Genet.* **6**, 881–892 (2005). 16341069 10.1038/nrg1723

[26] Rux, D. R. & Wellik, D. M. Hox genes in the adult skeleton: novel functions beyond embryonic development. *Dev. Dyn.* **246**, 310–317 (2017). 28026082 10.1002/dvdy.24482 PMC5508556

[27] Seifert, A., Werheid, D. F., Knapp, S. M. & Tobiasch, E. Role of Hox genes in stem cell differentiation. *World J. Stem Cells* **7**, 583 (2015). 25914765 10.4252/wjsc.v7.i3.583 PMC4404393

[28] Lin, X. iangsheng et al. Hoxa1 and Hoxa13 facilitate slow-twitch muscle formaiton in C2C12 cells and indirectly affect the lipid deposition of 3T3-L1 cells. *Anim. Sci. J.* **92**, e13544 (2021). 33738916 10.1111/asj.13544

[29] Seale, P. et al. Pax7 is required for the specification of myogenic satellite cells. *Cell* **102**, 777–786 (2000). 11030621 10.1016/s0092-8674(00)00066-0

[30] Cornelison, D. & Wold, B. J. Single-cell analysis of regulatory gene expression in quiescent and activated mouse skeletal muscle satellite cells. *Dev. Biol.* **191**, 270–283 (1997). 9398440 10.1006/dbio.1997.8721

[31] Asakura, A., Rudnicki, M. A. & Komaki, M. Muscle satellite cells are multipotential stem cells that exhibit myogenic, osteogenic, and adipogenic differentiation. *Differentiation* **68**, 245–253 (2001). 11776477 10.1046/j.1432-0436.2001.680412.x

[32] Dusterhöft, S., Yablonka-Reuveni, Z. & Pette, D. Characterization of myosin isoforms in satellite cell cultures from adult rat diaphragm, soleus and tibialis anterior muscles. *Differentiation* **45**, 185–191 (1990). 2090520 10.1111/j.1432-0436.1990.tb00472.x PMC4096307

[33] Lagord, C. et al. Differential myogenicity of satellite cells isolated from extensor digitorum longus (EDL) and soleus rat muscles revealed in vitro. *Cell Tissue Res.* **291**, 455–468 (1998). 9477302 10.1007/s004410051015

[34] Martelly, I. et al. Differential expression of FGF receptors and of myogenic regulatory factors in primary cultures of satellite cells originating from fast (EDL) and slow (Soleus) twitch rat muscles. *Cell. Mol. Biol.* **46**, 1239–1248 (2000). 11075953

[35] Ono, Y. et al. Slow-dividing satellite cells retain long-term self-renewal ability in adult muscle. *J. Cell Sci.* **125**, 1309–1317 (2012). 22349695 10.1242/jcs.096198

[36] Perruchot, M.-H., Ecolan, P., Sorensen, I. L., Oksbjerg, N. & Lefaucheur, L. In vitro characterization of proliferation and differentiation of pig satellite cells. *Differentiation* **84**, 322–329 (2012). 23023068 10.1016/j.diff.2012.08.001

[37] Queeno, S. R., Sterner, K. N. & O’Neill, M. C. Meta-analysis data of skeletal muscle slow fiber content across mammalian species. *Data Brief.* **50**, 109520 (2023). 37701714 10.1016/j.dib.2023.109520 PMC10493253

[38] Karlsson, A. H., Klont, R. E. & Fernandez, X. Skeletal muscle fibres as factors for pork quality. *Livest. Prod. Sci.* **60**, 255–269 (1999).

[39] Essén-Gustavsson, B. & Fjelkner-Modig, S. Skeletal muscle characteristics in different breeds of pigs in relation to sensory properties of meat. *Meat Sci.* **13**, 33–47 (1985). 22055444 10.1016/S0309-1740(85)80003-6

[40] Schiaffino, S. & Reggiani, C. Fiber types in mammalian skeletal muscles. *Physiol. Rev.* **91**, 1447–1531 (2011). 22013216 10.1152/physrev.00031.2010

[41] Lefaucheur, L. A second look into fibre typing–relation to meat quality. *Meat Sci.* **84**, 257–270 (2010). 20374784 10.1016/j.meatsci.2009.05.004

[42] Miao, X., Hastie, M., Ha, M. & Warner, R. Consumer response to blended beef burgers and chicken nuggets is influenced by ingredient and nutrition claims-qualitative assessment. *Future Foods* **8**, 100247 (2023).

[43] Masih, J. Understanding health-foods consumer perception using big data analytics. *J. Manag. Inf. Decis. Sci.* **24**, 1–15 (2021).

[44] de, L. as, Heras-Saldana, S., Chung, K. Y., Lee, S. H. & Gondro, C. Gene expression of Hanwoo satellite cell differentiation in longissimus dorsi and semimembranosus. *BMC Genom.* **20**, 1–15 (2019). 10.1186/s12864-019-5530-7 PMC6390542 30808286

[45] Evano, B. et al. Transcriptome and epigenome diversity and plasticity of muscle stem cells following transplantation. *PLoS Genet.* **16**, e1009022 (2020). 33125370 10.1371/journal.pgen.1009022 PMC7657492

[46] Grieshammer, U., Sassoon, D. & Rosenthal, N. A transgene target for positional regulators marks early rostrocaudal specification of myogenic lineages. *Cell* **69**, 79–93 (1992). 1313337 10.1016/0092-8674(92)90120-2

[47] Jin, L. et al. A pig BodyMap transcriptome reveals diverse tissue physiologies and evolutionary dynamics of transcription. *Nat. Commun.* **12**, 3715 (2021). 34140474 10.1038/s41467-021-23560-8 PMC8211698

[48] Yoshioka, K. et al. Hoxa10 mediates positional memory to govern stem cell function in adult skeletal muscle. *Sci. Adv.* **7**, eabd7924 (2021). 34108202 10.1126/sciadv.abd7924 PMC8189581

[49] Burke, A. C., Nelson, C. E., Morgan, B. A. & Tabin, C. Hox genes and the evolution of vertebrate axial morphology. *Development* **121**, 333–346 (1995). 7768176 10.1242/dev.121.2.333

[50] Houghton, L. & Rosenthal, N. Regulation of a muscle-specific transgene by persistent expression of Hox genes in postnatal murine limb muscle. *Dev. Dyn.* **216**, 385–397 (1999). 10633858 10.1002/(SICI)1097-0177(199912)216:4/5<385::AID-DVDY7>3.0.CO;2-G

[51] Yamamoto, M. & Kuroiwa, A. Hoxa-11 and Hoxa-13 are involved in repression of MyoD during limb muscle development. *Dev. growth Differ.* **45**, 485–498 (2003). 14706073 10.1111/j.1440-169x.2003.00715.x

[52] Poliacikova, G., Maurel-Zaffran, C., Graba, Y. & Saurin, A. J. Hox proteins in the regulation of muscle development. *Front. Cell Dev. Biol.* **9**, 731996 (2021). 34733846 10.3389/fcell.2021.731996 PMC8558437

[53] Schwoerer, S. et al. Epigenetic stress responses induce muscle stem-cell ageing by Hoxa9 developmental signals. *Nature* **540**, 428–432 (2016). 27919074 10.1038/nature20603 PMC5415306

[54] Dhar, G. A., Saha, S., Mitra, P. & Nag Chaudhuri, R. DNA methylation and regulation of gene expression: guardian of our health. *Nucleus* **64**, 259–270 (2021). 10.1007/s13237-021-00367-y PMC8366481 34421129

[55] Bonder, M. J. et al. Genetic and epigenetic regulation of gene expression in fetal and adult human livers. *BMC genom.* **15**, 1–13 (2014). 10.1186/1471-2164-15-860 PMC4287518 25282492

[56] Beckman, W. F., Jiménez, M.ÁL. & Verschure, P. J. Transcription bursting and epigenetic plasticity: an updated view. *Epigenetics Commun.* **1**, 6 (2021).

[57] Relaix, F. et al. Six homeoproteins directly activate Myod expression in the gene regulatory networks that control early myogenesis. *PLoS Genet.* **9**, e1003425 (2013). 23637613 10.1371/journal.pgen.1003425 PMC3636133

[58] Imbriano, C. & Molinari, S. Alternative splicing of transcription factors genes in muscle physiology and pathology. *Genes* **9**, 107 (2018). 29463057 10.3390/genes9020107 PMC5852603

[59] Giordani, J. et al. Six proteins regulate the activation of Myf5 expression in embryonic mouse limbs. *Proc. Natl. Acad. Sci. USA* **104**, 11310–11315 (2007). 17592144 10.1073/pnas.0611299104 PMC2040895

[60] Rodriguez-Esteban, C. et al. The T-box genes Tbx4 and Tbx5 regulate limb outgrowth and identity. *Nature* **398**, 814–818 (1999). 10235264 10.1038/19769

[61] Naiche, L. & Papaioannou, V. E. Loss of Tbx4 blocks hindlimb development and affects vascularization and fusion of the allantois. *Development* **130**, 2681–93 (2003). 10.1242/dev.00504 12736212

[62] Chao, Z. et al. Molecular characterization and expression patterns of Lbx1 in porcine skeletal muscle. *Mol. Biol. Rep.* **38**, 3983–3991 (2011). 21107715 10.1007/s11033-010-0516-1

[63] Walker, J. T., Flynn, L. E. & Hamilton, D. W. Lineage tracing of Foxd1-expressing embryonic progenitors to assess the role of divergent embryonic lineages on adult dermal fibroblast function. *FASEB BioAdvances* **3**, 541 (2021). 34258523 10.1096/fba.2020-00110 PMC8255845

[64] Daston, G., Lamar, E., Olivier, M. & Goulding, M. Pax-3 is necessary for migration but not differentiation of limb muscle precursors in the mouse. *Development* **122**, 1017–1027 (1996). 8631247 10.1242/dev.122.3.1017

[65] Relaix, F. et al. Pax3 and Pax7 have distinct and overlapping functions in adult muscle progenitor cells. *J. Cell Biol.* **172**, 91–102 (2006). 16380438 10.1083/jcb.200508044 PMC2063537

[66] Günther, S. et al. Myf5-positive satellite cells contribute to Pax7-dependent long-term maintenance of adult muscle stem cells. *Cell Stem Cell* **13**, 590–601 (2013). 23933088 10.1016/j.stem.2013.07.016 PMC4082715

[67] Mesires, N. T. & Doumit, M. E. Satellite cell proliferation and differentiation during postnatal growth of porcine skeletal muscle. *Am. J. Physiol. Cell Physiol.* **282**, C899–C906 (2002). 11880278 10.1152/ajpcell.00341.2001

[68] Park, J., Choi, H. & Shim, K. Inhibition of GSK3β promotes proliferation and suppresses apoptosis of porcine muscle satellite cells. *Animals* **12**, 3328 (2022). 36496849 10.3390/ani12233328 PMC9738253

[69] Dobin, A. et al. STAR: ultrafast universal RNA-seq aligner. *Bioinformatics* **29**, 15–21 (2013). 23104886 10.1093/bioinformatics/bts635 PMC3530905

[70] Zhu, Q., Fisher, S. A., Shallcross, J. & Kim, J. VERSE: a versatile and efficient RNA-Seq read counting tool. *bioRxiv* 053306 (2016).

[71] Love, M. I., Huber, W. & Anders, S. Moderated estimation of fold change and dispersion for RNA-seq data with DESeq2. *Genome Biol.* **15**, 1–21 (2014). 10.1186/s13059-014-0550-8 PMC4302049 25516281

[72] Wei, T. & Simko, V. *R package “corrplot”: Visualization of a Correlation Matrix* (Version 0.84) (Vienna, 2017).

[73] Wickham, H. & Sievert, C. *ggplot2: elegant graphics for data analysis*. Vol. 10 (Springer, 2009).

[74] Warnes, G. R. et al. gplots: various R programming tools for plotting data. *R package version 3.* https://CRAN.R-project.org/package=gplots (2016).

[75] Huang, D. W., Sherman, B. T. & Lempicki, R. A. Systematic and integrative analysis of large gene lists using DAVID bioinformatics resources. *Nat. Protoc.* **4**, 44–57 (2009). 19131956 10.1038/nprot.2008.211

