# Supplementary material for: Optimizing muscle satellite cell sources for cultured meat: anatomical origin influences cellular properties and quality attributes
Source: NPJ Sci Food. 2026 Jan 14;10:56. doi: 10.1038/s41538-026-00706-w (PMC12901286; doi:10.1038/s41538-026-00706-w)
Supplement: Supplementary file 1 — Supplementary materials_pdf [file 41538_2026_706_MOESM1_ESM.pdf]

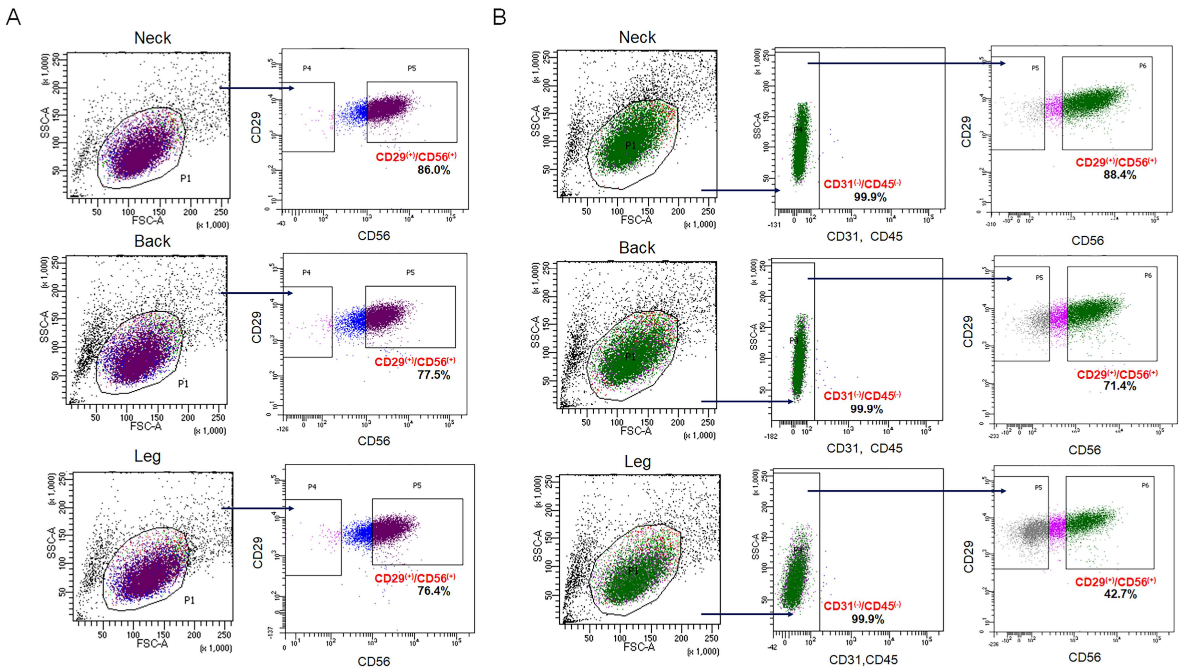


**Fig. S1. Comparison of PMSCs purification from different anatomical regions in two LYD pigs.** (A) The PMSCs were gated by CD29 and CD56. (B) The SCs were gated by CD31, CD45, CD29, and CD56. SSC, side scatter; FSC, forward scatter.

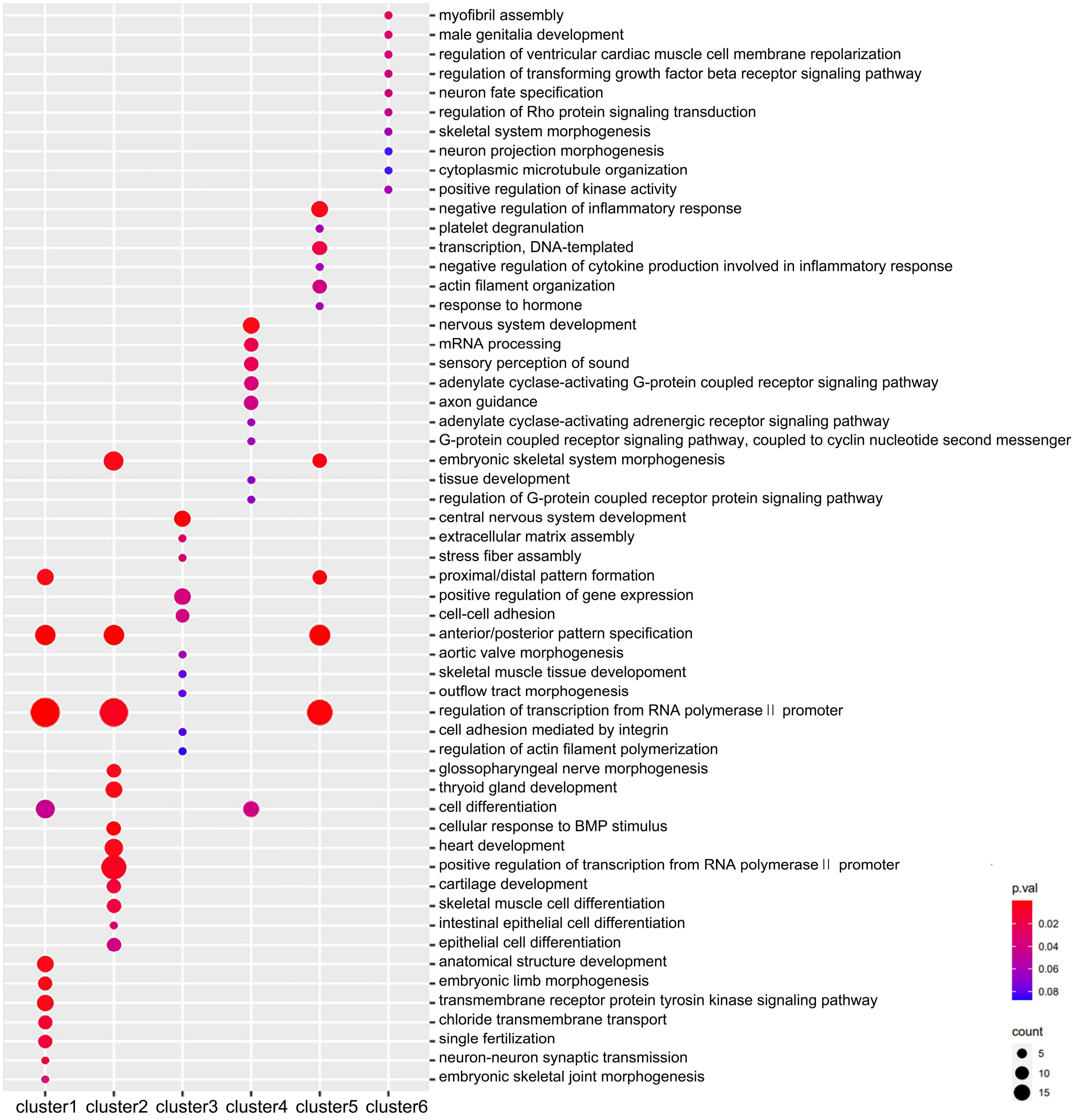


**Fig. S2. Characteristics of the neck, back, and leg-derived PMSCs gene expression.** Gene ontology biological process (GOBP) for all clusters was visualized through dot plots. $p < 0.05$, FC $\geq 3$.
